# Supplementary material for: Significance Tests of Feature Relevance for a Black-Box Learner
Source: IEEE Trans Neural Netw Learn Syst. Author manuscript; Available in PMC 2024 Mar 6. (PMC10915654; doi:10.1109/TNNLS.2022.3185742)
Supplement: supp1-3185742 [file NIHMS1964699-supplement-supp1-3185742.pdf]

Supplementary Materials for "Significance Tests of Feature Relevance for a Black-Box Learner"

Ben Dai, Xiaotong Shen, and Wei Pan

APPENDIX A
TWO-SPLIT TEST

To treat the high *bias-sd-ratio* issue as described in Section 2, we propose an alternative of the one-split test by further splitting an inference sample into two equal subsamples yet the perturbation is not required. For simplicity, we assume m is an even number.

A. Two-split test

Given $(\widehat{f}_n, \widehat{g}_n)$, we evaluate them based on these two independent subsamples to yield our two-split test statistic:

$$\Lambda_n^{(2)} = \frac{\sum_{j=1}^{m/2} \Delta_{n,j}^{(2)}}{\sqrt{\frac{m}{2}}\widehat{\sigma}_n^{(2)}}, \tag{A.1}$$

$$\Delta_{n,j}^{(2)} = l(\widehat{f}_n(\boldsymbol{X}_{n+j}), \boldsymbol{Y}_{n+j}) - l(\widehat{g}_n(\boldsymbol{Z}_{n+m+j}), \boldsymbol{Y}_{n+m+j}),$$

where $\widehat{\sigma}_n^{(2)}$ is the sample standard deviation of $\{\Delta_{n,j}^{(2)}\}_{j=1}^{m/2}$ given $\widehat{f}_n$ and $\widehat{g}_n$. In this fashion, perturbation is no longer required.

Similarly, the two-split test proceeds as the one-split test except that its p-value is computed as $P^{(2)} = \Phi(\Lambda_n^{(2)})$ based on Theorem A.1.

To derive the asymptotic null distribution of $\Lambda_n^{(2)}$, we make the parallel assumptions B$'$ and C$'$.

Assumption B$'$ (Lyapounov condition for $\Lambda_n^{(2)}$). Assume that

$$m^{-\mu}\mathbb{E}\big(|\Delta_{n,1}^{(2)}|^{2(1+\mu)}\big|\mathcal{E}_n\big) \xrightarrow{p} 0, \quad \text{as } n \to \infty$$

for some constant $\mu > 0$, where $\Delta_{n,1}^{(2)}$ is defined in (A.1) and $\xrightarrow{p}$ denotes convergence in probability.

Assumption C$'$ (Variation) Assume that $\text{Var}(\Delta_{n,1}^{(2)}|\mathcal{E}_n) \xrightarrow{p} (\sigma^{(2)})^2$ as $n \to \infty$.

Under some mild assumptions, $(\sigma^{(2)})^2 = \text{Var}\big(l(f^*(\boldsymbol{X}), \boldsymbol{Y})\big) + \text{Var}\big(l(g^*(\boldsymbol{Z}(\boldsymbol{X})), \boldsymbol{Y})\big) > 0$, c.f., Lemma C.1.

Theorem A.1 (Asymptotic null distribution): In addition to Assumptions A, B$'$, and C$'$, if $m = o(n^{2\gamma})$, then under H_0,

$$\Lambda_n^{(2)} \xrightarrow{d} N(0, 1), \quad \text{as} \quad n \to \infty. \tag{A.2}$$

Furthermore, the p-value $\bar{P}^{(2)}$ for the combined two-split test can be defined exactly as in Section 2.3.

Theorem A.2 (Type I error for the combined two-split test): Suppose that Assumption A and B'-C' are satisfied for the two-split test (A.1), if $m = o(n^{2\gamma})$, then under H_0, for any $0 < \alpha < 1$ and any $U \geq 2$,

$$\lim_{n\to\infty} \mathbb{P}\big(\bar{P}^{(2)} \leq \alpha | H_0\big) \leq \alpha,$$

where $\bar{P}^{(2)}$ is defined as the q-order test or the Hommel's test based on $(P_1^{(2)}, \cdots, P_U^{(2)})$.

B. Type II error for two-split tests

This section performs Type II error analysis of the two-split test in (A.1). Consider an alternative hypothesis H_a : $R(f^*) - R_{\mathcal{S}}(g^*) = -m^{-1/2}\delta < 0$ for $\delta > 0$. The Type II error of the two-split test in (A.1) and its combined tests can be written as

$$\beta_n^{(2)}(\delta) = \mathbb{P}(P^{(2)} \geq \alpha | H_a), \quad \bar{\beta}_n^{(2)}(\delta) = \mathbb{P}(\bar{P}^{(2)} \geq \alpha | H_a),$$

where $\mathbb{P}(\cdot|H_a)$ denotes the probability under H_a and $\alpha > 0$ is the nominal level.

Theorems A.3 and A.4 suggest that the Type II error of the two-split test and its combined test tend to zero as $\delta \to \infty$.

Theorem A.3 (Limiting Type II error of the two-split test): Under Assumption A, B' and C', if $m = o(n^{2\gamma})$, then we have

$$\lim_{n\to\infty} \sup \beta_n^{(2)}(\delta) = \Phi\Big(z_\alpha - \frac{\delta}{\sqrt{2}\sigma^{(2)}}\Big),$$
$$\lim_{\delta\to\infty} \lim_{n\to\infty} \sup \beta_n^{(2)}(\delta) = 0, \tag{A.3}$$

where $z_\alpha = \Phi^{-1}(1-\alpha)$ is the $100(1-\alpha)$th percentile of the standard normal distribution.

Theorem A.4 (Limiting Type II error of the combined two-split test): Under Assumption A, B$'$ and C$'$, $m = o(n^{2\gamma})$, then for $\bar{P}^{(2)}$ defined as the q-order combined test, we have

$$\lim_{n\to\infty} \sup \bar{\beta}_n^{(2)}(\delta) \leq \min\Big(\frac{U}{\alpha q}\Gamma, 1\Big),$$
$$\lim_{\delta\to\infty} \lim_{n\to\infty} \sup \bar{\beta}_n^{(2)}(\delta) = 0,$$

and for $\bar{P}^{(2)}$ defined as the Hommel combined test, we have

$$\lim_{n\to\infty} \sup \bar{\beta}_n^{(2)}(\delta) \leq \min\Big\{\frac{C_U U}{\alpha q}\Gamma, 1; q = 1, \cdots, U\Big\},$$
$$\lim_{\delta\to\infty} \lim_{n\to\infty} \sup \bar{\beta}_n^{(2)}(\delta) = 1,$$

where $\Gamma = \Phi\big(-\frac{\delta}{2\sigma^{(l)}}\big) + \sqrt{\frac{q-1}{U-q+1}}\Big(\Phi\big(\frac{\delta}{2\sigma^{(l)}}\big) - \Phi^2\big(\frac{\delta}{2\sigma}\big) - 2T(-\frac{\delta}{2\sigma^{(l)}}, \frac{\sqrt{3}}{3})\Big)^{1/2}$, and $T(h, a) = (2\pi)^{-1}\int_0^a \frac{\exp(-h^2(1+x^2)/2)}{x^2+1}dx$ is Owen's T function [1].

Ben Dai is with the Department of Statistics, The Chinese University of Hong Kong, Hong Kong SAR (email: bendai@cuhk.edu.hk).
Xiaotong Shen is with the School of Statistics, University of Minnesota, MN, 55455 USA (email: xshen@umn.edu).
Wei Pan is with the Division of Biostatistics, University of Minnesota, MN, 55455 USA. (email: panxx014@umn.edu).

C. Data-adaptive sample splitting for the two-split test

This section develops a computing scheme to determine the sample splitting ratio or ζ to achieve our objective of controlling Type I error in a finite-sample situation.

Type I error of the two-split test is $\text{Err}^{(2)}(\rho,\zeta)=\mathbb{P}(\Lambda_n^{(2)}\leq z_\alpha|H_0)$, which is a function of splitting ratio ζ:

$$\widehat{\text{Err}^{(2)}}(\zeta)=T^{-1}\sum_{t=1}^{T}\mathbb{I}\big(\Lambda_n^{(2,t)}\leq z_\alpha\big), \tag{A.4}$$

where $\Lambda_n^{(l,t)}$; $l=1,2$, computed based on the permuted estimation sample $\{\tilde{\boldsymbol{X}}_j^{(t)},\tilde{Y}_j^{(t)}\}_{j=1}^n$ and the permuted inference sample $\{\tilde{\boldsymbol{X}}_j^{(t)},\tilde{Y}_j^{(t)}\}_{i=n+1}^N$; $t=1,\cdots,T$.

Moreover, data-adaptive is also applicable to the two-split test:

$$\widehat{\zeta}=\min\{\zeta\in\boldsymbol{\zeta}:\widehat{\text{Err}^{(2)}}(\zeta)\leq\alpha\}, \tag{A.5}$$

where $\widehat{\text{Err}^{(2)}}(\zeta)$ is computed via (A.4). Furthermore, ζ for the combined tests are estimated by replacing $P^{(2)}$ by $\bar{P}^{(2)}$ based on permuted sample. The computational scheme of the proposed two-split tests is summarized in Algorithm 1.

Algorithm 1: Two-split test for feature relevance to prediction.

Input : Data: $(\boldsymbol{x}_i,\boldsymbol{y}_i)_{i=1}^N$; Set of hypothesized features: $\mathcal{S}$; Number of splitting: U
Output: p-value for the test in Section 2.

Estimate $\widehat{\zeta}$ from (A.5) ;
for $u=1,\cdots,U$ **do**
  Shuffle data;
  Split data into an estimation sample and an inference sample, where $m=\hat{\zeta}N$ and $n=N-m$;
  Generate dual samples via (1) for estimation/inference subsets;
  Compute $\Lambda_u^{(2)}$ from (A.1);
  Compute p-value $P_u^{(2)}=\Phi(\Lambda_u^{(2)})$
end
if $U>1$ **then** // combined two-split test
  Compute the combined p-value $\bar{P}^{(2)}$ via Section 2.3;
  return *p-value* $\bar{P}^{(2)}$
else // non-combined two-split test
  return *p-value* $P_1^{(2)}$
end

Appendix B
Additional numerical examples

A. Numerical results for simulated examples

The numerical results for Examples 3-6 are indicated in Tables B.1, B.2, B.3, and B.4, respectively.

B. Comparison with the likelihood ratio test for a non-blackbox learner

This subsection compares the proposed tests with the likelihood ratio test (LRT) for a logistic regression, although the former is designed for a blackbox learner. In simulations, we generate a random sample $(\boldsymbol{X}_i,Y_i)_{i=1}^N$ as follows. First, we generate a feature vector $\boldsymbol{X}$ and the regression parameter vector $\boldsymbol{\theta}$ from $N(\mathbf{0},\boldsymbol{I}_d)$. Second, we generate a binary response Y as $Y=\text{Sign}(\boldsymbol{\theta}^\intercal\boldsymbol{Z}+.1\epsilon)$ with $\boldsymbol{Z}_{1:10}=\mathbf{0}$ and $\boldsymbol{Z}_{11:100}=\boldsymbol{X}_{11:100}$. Now consider a hypothesis test in Section 2 to determine if $\boldsymbol{X}_\mathcal{S}$ is functionally relevant to the prediction of $\boldsymbol{Y}$, consider null H_0 and alternative H_a hypotheses in three cases: (i) $\mathcal{S}=\{1,\cdots,10\}$, (ii) $\mathcal{S}=\{5,\cdots,15\}$, (iii) $\mathcal{S}=\{10,\cdots,15\}$, where the sample size N is $500,800,1000$ and $d=100$.

For implementation, we fit a logistic regression model with sample $(\boldsymbol{X}_i,Y_i)_{i=1}^N$ via stochastic gradient descent with a learning rate 0.05 for LRT based on the Github repo[1]. For the proposed tests, we use the same fitting and splitting parameters for data-adaptive scheme as in Section 6.2.

As indicated in Table B.5, LRT and the proposed tests control the Type I error but the proposed tests exhibit a fair yet insubstantial amount of power loss. The loss of power of the proposed test is primarily due to their smaller inference sample.

C. Inflated Type I errors for holdout permutation test (HPT) and permutation test (PT)

This subsection demonstrates that HPT and PT can incur inflated Type I errors. Algorithm 2 summarizes the procedure for the permutation test.

Algorithm 2: Permutation test for feature relevance to prediction.

Input : Data $\mathcal{D}=(\boldsymbol{x}_i,\boldsymbol{y}_i)_{i=1}^N$; Set of hypothesized features: $\mathcal{S}$; Number of permutations T
Output: p-values for marginal independence

Compute the cross-validation score s_0 on data $\mathcal{D}$.
for $t=1,\cdots,T$ **do**
  Generate $\mathcal{D}_t$ by simultaneously permuting values of hypothesized features in H_0.
  Compute the cross-validation score s_t based on data $\mathcal{D}_t$
end
Compute p-value:

$$\widehat{p}=\frac{\big|\{s_t\leq s_0|t=1,\cdots,T\}\big|+1}{T+1}.$$

Specifically, we consider the same simulation setting in Section 4.2, with $N=2000$, $B=0.1$, $r=0.85$, $p=100$, $\tau=2$, $L=2$, $\varpi=128$, and $|\mathcal{S}_0|=3$. The Type I errors of the null hypothesis based on $\mathcal{S}=\{1,2,3\}$ for all tests are reported in Table B.6 over 100 simulations. As indicated in Table B.6, the one-split/two-split tests and their combined tests

[1]https://gist.github.com/rnowling/ec9c9038e492d55ffae2ae257aa4acd9

Test	L	#Parameters	width ϖ	Type I error	Power (1 - Type II error)	Time (Second)
One-split	2	3232	32	0.050	(0.26, 0.82, 0.84)	36.91(0.21)
		6464	64	0.041	(0.27, 0.84, 0.89)	35.48(0.17)
		12928	128	0.048	(0.22, 0.90, 0.91)	35.31(0.18)
	3	4256	32	0.054	(0.66, 1.00, 1.00)	39.44(0.29)
		10560	64	0.051	(0.65, 1.00, 1.00)	38.41(0.24)
		29312	128	0.050	(0.61, 0.99, 1.00)	41.24(0.29)
	4	5280	32	0.066	(0.98, 1.00, 1.00)	42.27(0.22)
		18752	64	0.048	(1.00, 1.00, 1.00)	41.81(0.30)
		62080	128	0.054	(0.99, 1.00, 1.00)	43.12(0.43)
Two-split	2	3232	32	0.050	(0.03, 0.16, 0.13)	35.76(0.20)
		6464	64	0.050	(0.04, 0.11, 0.13)	35.28(0.30)
		12928	128	0.055	(0.05, 0.14, 0.16)	33.65(0.16)
	3	4256	32	0.044	(0.19, 0.63, 0.63)	37.19(0.20)
		10560	64	0.035	(0.16, 0.47, 0.62)	35.82(0.20)
		29312	128	0.035	(0.18, 0.51, 0.58)	37.02(0.16)
	4	5280	32	0.044	(0.54, 1.00, 1.00)	43.54(0.34)
		18752	64	0.041	(0.54, 1.00, 1.00)	40.28(0.26)
		62080	128	0.045	(0.54, 1.00, 1.00)	47.07(0.31)
Comb. one-split	2	3232	32	0.046	(0.39, 0.98, 0.94)	61.99(0.17)
		6464	64	0.022	(0.22, 0.97, 0.97)	61.40(0.16)
		12928	128	0.020	(0.40, 1.00, 1.00)	60.42(0.45)
	3	4256	32	0.049	(0.80, 1.00, 1.00)	63.71(0.21)
		10560	64	0.032	(0.79, 1.00, 1.00)	65.23(0.14)
		29312	128	0.046	(0.86, 1.00, 1.00)	68.28(0.26)
	4	5280	32	0.054	(1.00, 1.00, 1.00)	145.38(0.73)
		18752	64	0.032	(1.00, 1.00, 1.00)	112.77(1.15)
		62080	128	0.032	(1.00, 1.00, 1.00)	127.07(0.79)
Comb. two-split	2	3232	32	0.022	(0.00, 0.08, 0.11)	30.32(0.12)
		18752	64	0.015	(0.06, 0.09, 0.15)	31.73(0.10)
		12928	128	0.017	(0.04, 0.13, 0.08)	33.44(0.12)
	3	4256	32	0.011	(0.08, 0.75, 0.76)	41.46(0.34)
		10560	64	0.007	(0.08, 0.66, 0.67)	34.27(0.27)
		29312	128	0.030	(0.10, 0.70, 0.65)	76.28(0.54)
	4	4256	32	0.013	(0.65, 1.00, 1.00)	48.99(0.61)
		18752	64	0.025	(0.65, 1.00, 1.00)	39.13(0.07)
		62080	128	0.014	(0.69, 1.00, 1.00)	49.05(0.15)

TABLE B.1: Type I errors and powers of the one-split and two-split tests and their combined tests in Example 3 at a nominal level $\alpha = 0.05$.

TABLE B.2: Type I errors and powers of the one-split and two-split tests and their combined tests in Example 4 at a nominal level $\alpha = 0.05$. The data-adaptive tuning scheme is applied.

Test	$\lvert\mathcal{S}_0\rvert$	Type I error	Power(1 - Type II error)
One-split	3	0.047	(0.28, 0.95, 0.96)
	5	0.050	(0.61, 0.99, 1.00)
	10	0.037	(1.00, 1.00, 1.00)
Two-split	3	0.052	(0.09, 0.19, 0.31)
	5	0.035	(0.18, 0.51, 0.58)
	10	0.042	(0.59, 0.95, 0.98)
Comb. one-split	3	0.035	(0.24, 1.00, 1.00)
	5	0.046	(0.86, 1.00, 1.00)
	10	0.019	(1.00, 1.00, 1.00)
Comb. two-split	3	0.020	(0.05, 0.20, 0.19)
	5	0.030	(0.10, 0.70, 0.65)
	10	0.013	(0.72, 1.00, 1.00)

TABLE B.3: Empirical Type I errors and powers of the one-split and two-split tests and their combined tests in Example 5 at a nominal level $\alpha = 0.05$. The data-adaptive tuning scheme is applied.

Test	r	Type I error	Power(1 - Type II error)
One-split	0.00	0.044	(0.55, 0.98, 0.96)
	0.25	0.050	(0.61, 0.99, 1.00)
	0.50	0.052	(0.89, 1.00, 1.00)
Two-split	0.00	0.040	(0.09, 0.32, 0.35)
	0.25	0.035	(0.18, 0.51, 0.58)
	0.50	0.039	(0.09, 0.80, 0.79)
Comb. one-split	0.00	0.029	(0.64, 1.00, 1.00)
	0.25	0.046	(0.86, 1.00, 1.00)
	0.50	0.033	(0.98, 1.00, 1.00)
Comb. two-split	0.00	0.018	(0.04, 0.38, 0.31)
	0.25	0.030	(0.10, 0.70, 0.65)
	0.50	0.022	(0.09, 0.95, 0.98)

control Type I error, yet neither HPT nor PT could control the Type I error under a nominal level.

D. Effect of size of grids of data-adaptive splitting scheme

This subsection demonstrates the effect of size of grids of the perturbation size ρ and the splitting ratio ζ for the heuristic

TABLE B.4: Empirical Type I errors and powers of the different combined methods for the one-split test in **Example 6** at a nominal level $\alpha = 0.05$.

Test	B	(L, d)	Comb. method	Type I error	Power(1 - Type II error)
Comb. one-split	0.20	(3, 128)	Hommel	0.019	(0.27, 0.93, 0.93)
			Bonferroni	0.044	(0.43, 0.95, 0.98)
			1st-Quantile	0.004	(0.13, 0.89, 0.95)
			median	0.000	(0.02, 0.69, 0.75)
			Cauchy	0.050	(0.41, 1.00, 1.00)
			harmonic	0.014	(0.20, 0.84, 0.94)
Comb. one-split	0.40	(4, 32)	Hommel	0.054	(1.00, 1.00, 1.00)
			Bonferroni	0.097	(1.00, 1.00, 1.00)
			1st-Quantile	0.011	(1.00, 1.00, 1.00)
			median	0.000	(1.00, 1.00, 1.00)
			Cauchy	0.099	(1.00, 1.00, 1.00)
			harmonic	0.035	(1.00, 1.00, 1.00)

TABLE B.5: Empirical Type I errors and powers of the likelihood ratio test, the one-split and two-split tests, and their combined tests at a nominal level $\alpha = 0.05$. The likelihood ratio uses the asymptotic χ-square distribution for the null distribution.

Test	sample size	Type I error	Power(1 - Type II error)
One-split	500	0.040	(0.15, 0.20)
	800	0.020	(0.42, 0.44)
	1000	0.040	(0.69, 0.77)
Two-split	500	0.032	(0.13, 0.13)
	800	0.030	(0.18, 0.18)
	1000	0.020	(0.39, 0.34)
Comb. one-split	500	0.042	(0.19, 0.24)
	800	0.010	(0.53, 0.59)
	1000	0.019	(0.79, 0.85)
Comb. two-split	500	0.020	(0.06, 0.15)
	800	0.012	(0.32, 0.37)
	1000	0.002	(0.51, 0.50)
LRT	500	0.003	(0.52, 0.55)
	800	0.000	(0.92, 0.93)
	1000	0.035	(0.98, 0.99)

data-adaptive scheme in Section 5.2.

For illustration, we consider the same simulation setting as in Example 1 with $N = 6000$, then the grids $\boldsymbol{\zeta} = \{.2, .6\}, \{.2, .4, .6, .8\}, \{.2, .3, \cdots, .9\}$, and $\boldsymbol{\rho} = \{.01, .1, 1\}, \{.01, .05, .1, .5, 1\}$ are examined. The Type I error and power functions for the proposed methods are summarized in Table B.7.

As indicated in Table B.7, Type I error, power and computation time based on data adaptive splitting method do not significantly affected by the grid sets of (ζ, ρ) due to the early stopping mechanism.

E. Simulation for model misspecification

This subsection examines performance for the proposed tests in situation that the true regression function f^* belongs to a bigger class than a neural network class $\mathcal{H}$. Toward this end, we simulate random samples $(\boldsymbol{X}_i, Y_i)_{i=1}^N$ as follows. First, we simulate $\boldsymbol{X}_i$ from $N(\mathbf{0}, \boldsymbol{I}_d)$ with $d = 10$. Second, we generate Y_i:

$$Y_i = 0.1X_{i6} + 0.2X_{i7}^2 + 0.3X_{i8}^3 + 0.4X_{i9}X_{i10} + 0.3\epsilon,$$

where $\epsilon \sim N(0, 1)$. Now consider the null hypothesis in (1) to determine if $\boldsymbol{X}_\mathcal{S}$ is functionally relevant to the prediction of Y with the true null H_0 and alternative H_a hypotheses in three cases: (i) $\mathcal{S} = \{1, \cdots, 5\}$, (ii) $\mathcal{S} = \{3, \cdots, 7\}$, (iii) $\mathcal{S} = \{6, 7, 8\}$, where the sample size N is 1000, 2000, 6000. Note that (i) is for Type I error analysis and (ii)-(iv) are for power analysis.

As indicated in Table B.8, the proposed tests control the Type I errors for all different sample sizes, and the power increases as the sample size becoming larger. The numerical results also confirm the theoretical analysis in Section C.

Appendix C
Theoretical example

This section provides a specific theoretical example to illustrate the one-split test, and verify Assumptions A-C. Consider nonparametric regression,

$$Y = f^*(\boldsymbol{X}) + \epsilon, \quad \epsilon \sim N(0, \varsigma^2), \tag{C.1}$$

where $f^*(\boldsymbol{x})$ is an unknown function on $\boldsymbol{x} \in [-1, 1]^d$. It is known that $f^*(\boldsymbol{x}) = g^*(\boldsymbol{z})$ only depends on a subset of features of $\boldsymbol{x}$, in which $\boldsymbol{z}_{\mathcal{S}_0} = \mathbf{0}$ and $\boldsymbol{z}_{\mathcal{S}_0^c} = \boldsymbol{x}_{\mathcal{S}_0^c}$ with $\mathcal{S}_0 = \{1, \cdots, |\mathcal{S}_0|\}$. Given a hypothesized index set $\mathcal{S}$, our goal is to test if $\boldsymbol{X}_\mathcal{S}$ is relevant to predicting the outcome Y, as specified in Section 2.

For illustration, consider $f^*(\boldsymbol{x}) = A\big((\boldsymbol{W}^L)^* A\big((\boldsymbol{W}^{L-1})^* \cdots A((\boldsymbol{W}^1)^*\boldsymbol{x})\big)\big)$, where $A(\cdot)$ is the ReLU activation function, $(\boldsymbol{W}^l)^* = ((w_{ij}^l)^*) \in \mathbb{R}^{d_l \times d_{l-1}}$ is a weight matrix, $\|(\boldsymbol{w}_j^l)^*\|_2 = \tau / d_{l-1}^{1/2}$, $(\boldsymbol{w}_j^l)^*$ is the j-th column of the matrix $(\boldsymbol{W}^l)^*$, $\tau > 0$ is a constant, d_l is the width for the l-th layer, and $d_0 = d$, $d_L = 1$, $d_1 = \cdots = d_{L-1} = \varpi$ and L is the depth of the network. Clearly, $f^* \in \mathcal{H}$, where $\mathcal{H}$ is defined as:

$$\mathcal{H} = \{f(\boldsymbol{x}) = A\big(\boldsymbol{W}^L A\big(\boldsymbol{W}^{L-1} \cdots A(\boldsymbol{W}^1\boldsymbol{x})\big)\big) : \|\boldsymbol{W}^l\|_2 \le \tau, \|\boldsymbol{W}^l\|_{2,1} \le \tau\}.$$

Given an estimation sample $(\boldsymbol{X}_i, Y_i)_{i=1}^n$ and an inference sample $(\boldsymbol{X}_j, Y_j)_{j=n+1}^{n+m}$, consider a loss function $l(\hat{y}, y) = (\hat{y}-$

TABLE B.6: Type I errors of the holdout permutation test (HPT), the permutation test (PT), the one-split and two-split tests and their combined tests at a nominal level $\alpha = 0.05$.

One-split	Two-split	Comb. one-split	Comb. two-split	HPT	PT
0.03	0.04	0.01	0.01	0.12	0.97

TABLE B.7: Empirical Type I errors and powers of the one-split and two-split tests with different size of grids at a nominal level $\alpha = 0.05$.

Test	$\boldsymbol{\zeta}$	$\boldsymbol{\rho}$	Type I error	Power(1 - Type II error)	Time (Second)
One-split	{.2, .6}	{.01, .1, 1}	0.020	(0.65, 1.00, 1.00)	31.4(1.2)
		{.01, .05, .1, .5, 1}	0.022	(0.58, 1.00, 1.00)	30.4(1.1)
	{.2, .4, .6, .8}	{.01, .1, 1}	0.010	(0.62, 1.00, 1.00)	29.4(0.5)
		{.01, .05, .1, .5, 1}	0.050	(0.61, 0.99, 1.00)	41.2(0.3)
	{.2, .3, $\cdots$, .9}	{.01, .1, 1}	0.009	(0.63, 1.00, 1.00)	32.2(2.0)
		{.01, .05, .1, .5, 1}	0.021	(0.70, 1.00, 1.00)	32.1(1.2)
Two-split	{.2, .6}	–	0.032	(0.17, 0.49, 0.56)	30.6(0.6)
	{.2, .4, .6, .8}	–	0.035	(0.18, 0.51, 0.58)	37.0(0.2)
	{.2, .3, $\cdots$, .9}	–	0.031	(0.16, 0.58, 0.62)	30.3(0.7)

TABLE B.8: Empirical Type I errors and powers of the one-split, two-split tests, and their combined tests in the misspecified situation at a nominal level $\alpha = 0.05$. The data-adaptive scheme is applied to determine the splitting ratio and perturbation size.

Test	Sample size	Type I error	Power(1 - Type II error)
One-split	1000	0.000	(0.11, 1.00)
	2000	0.000	(0.56, 1.00)
	6000	0.002	(0.97, 1.00)
Two-split	1000	0.000	(0.02, 0.77)
	2000	0.001	(0.33, 0.95)
	6000	0.001	(0.94, 1.00)
Comb. one-split	1000	0.028	(0.15, 1.00)
	2000	0.000	(0.79, 1.00)
	6000	0.001	(1.00, 1.00)
Comb. two-split	1000	0.000	(0.02, 0.82)
	2000	0.002	(0.33, 1.00)
	6000	0.002	(1.00, 1.00)

$y)^2$, where $\hat{y}$ is the predicted outcome of y and the prediction functions $(\widehat{f}_n, \widehat{g}_n)$ are obtained:

$$\widehat{f}_n = \underset{f \in \mathcal{H}}{\text{argmin}} \; n^{-1} \sum_{i=1}^{n} l\big(f(\boldsymbol{X}_i), Y_i\big);$$

$$\widehat{g}_n = \underset{g \in \mathcal{H}}{\text{argmin}} \; n^{-1} \sum_{i=1}^{n} l\big(g(\boldsymbol{Z}_i), Y_i\big). \tag{C.2}$$

To solve (C.2), we apply a stochastic gradient descent (SGD) algorithm. In general, SGD finds a local minimum of a nonconvex objective function [2] but a global minimizer in some special situations [3], [4].

Lemma C.1 is a version of Theorems 2 and 4, leading to the desired asymptotic null distribution and power of the one-split test in this specific example.

Lemma C.1: If $m = o(n^{2\gamma})$ with $\gamma = 1 - \omega$ for any $\omega > 0$, then the one-split test based on $\widehat{f}_n$ and $\widehat{g}_n$ from (C.2) satisfies: under H_0,

$$\lim_{n\to\infty} \mathbb{P}\big(P^{(1)} \leq \alpha | H_0\big) = \alpha, \quad \lim_{n\to\infty} \mathbb{P}\big(\bar{P}^{(1)} \leq \alpha | H_0\big) \leq \alpha.$$

Under H_a,

$$\lim_{\delta\to\infty} \lim_{n\to\infty} \sup \beta_n(\delta) = 0, \qquad \lim_{\delta\to\infty} \lim_{n\to\infty} \sup \bar{\beta}_n(\delta) = 0.$$

As a remark, we note that Lemma C.1 can be extended to a misspecified model situation, where $f^* \notin \mathcal{H}$ but belongs to a larger space such as $\mathcal{C}_d^{\xi}$, the ξ-Hölder functional space. In such a situation, the approximation error of f^* by $\mathcal{H}$ plays a role in the rate of convergence. Still, the rate in Assumption A can be obtained; for instance, the rate is $n^{-\xi/(2\xi+d+5)}$ for a neural net with one hidden layer [5], and the rate is $n^{-2\xi/(2\xi+d)}(\log n)$ for a two-layer neural net with the sigmoid activation function [6], [7]. Moreover, the numerical experiment for a misspecified model situation is illustrated in Section B-E.

Appendix D
Technical proofs

In this section, we rewrite m as m_n to emphasize the monotonicity of m as a subsequence of n, that is, $m_1 < \cdots < m_n$. **Proof of Lemma 1.** We first prove that $\boldsymbol{Y} \perp \boldsymbol{X}_{\mathcal{S}} \mid \boldsymbol{X}_{\mathcal{S}^c}$ yields $R(f^*) - R_{\mathcal{S}}(g^*) = 0$. To find f^* and g^*, it suffices to consider the pointwise minimization of $R(f)$ and $R_{\mathcal{S}}(g)$, that is, for any $\boldsymbol{x}$, we have

$$\begin{aligned} f^*(\boldsymbol{x}) &= \underset{u}{\text{argmin}} \, \mathbb{E}\big(l(u, \boldsymbol{Y}) | \boldsymbol{X}_{\mathcal{S}} = \boldsymbol{x}_{\mathcal{S}}, \boldsymbol{X}_{\mathcal{S}^c} = \boldsymbol{x}_{\mathcal{S}^c}\big) \\ &= \underset{u}{\text{argmin}} \, \mathbb{E}\big(l(u, \boldsymbol{Y}) | \boldsymbol{X}_{\mathcal{S}^c} = \boldsymbol{x}_{\mathcal{S}^c}\big) \\ &= \underset{u}{\text{argmin}} \, \mathbb{E}\big(l(u, \boldsymbol{Y}) | \boldsymbol{Z}(\boldsymbol{X}) = \boldsymbol{z}(\boldsymbol{x})\big) = g^*(\boldsymbol{z}(\boldsymbol{x})), \end{aligned}$$

where the second equality follows from the conditional independence. Therefore, $R(f^*) = \mathbb{E}\big(l(f^*(\boldsymbol{X}), \boldsymbol{Y})\big) = \mathbb{E}\big(l(g^*(\boldsymbol{Z}(\boldsymbol{X})), \boldsymbol{Y})\big) = R_{\mathcal{S}}(g^*)$.

Next, we show that H_0 is equivalent to conditional independence almost surely, if the cross-entropy loss $l(f(\boldsymbol{X}), Y) = -\boldsymbol{1}_Y^{\intercal} \log(f(\boldsymbol{X}))$ is used in Section 2. Note that $\boldsymbol{f}_k^*(\boldsymbol{x}) =$

$\mathbb{P}(Y=k|\boldsymbol{X}=\boldsymbol{x})$ and $\boldsymbol{g}_k^*(\boldsymbol{z}) = \mathbb{P}(Y=k|\boldsymbol{Z}(\boldsymbol{X})=\boldsymbol{z}(\boldsymbol{x}))$, we have

$$0 = R(f^*) - R_{\mathcal{S}}(g^*) = \mathbb{E}\Big(\mathbf{1}_Y^\intercal \log\big(\frac{\boldsymbol{f}^*(\boldsymbol{X})}{\boldsymbol{g}^*(\boldsymbol{Z}(\boldsymbol{X}))}\big)\Big) = \mathbb{E}\Big(\mathrm{KL}\big(\boldsymbol{f}^*(\boldsymbol{X}), \boldsymbol{g}^*(\boldsymbol{Z}(\boldsymbol{X}))\big)\Big),$$

which yields that $\mathrm{KL}\big(\boldsymbol{f}^*(\boldsymbol{X}), \boldsymbol{g}^*(\boldsymbol{Z}(\boldsymbol{X}))\big) = 0$ with probability one, and $\mathrm{KL}(\cdot,\cdot)$ is the Kullback–Leibler divergence. Thus, $\boldsymbol{f}^*(\boldsymbol{X}) = \boldsymbol{g}^*(\boldsymbol{Z}(\boldsymbol{X}))$ with probability one. This leads to the desirable results. □

Proof of Theorems 1 and Theorem A.1. Note that $\Lambda_n^{(l)} = T_{n,1}^{(l)} + T_{n,2}^{(l)} + T_{n,3}^{(l)}$, where $T_{n,1}^{(l)}$, $T_{n,2}^{(l)}$, and $T_{n,3}^{(l)}$ are defined as

$$T_{n,1}^{(l)} = \frac{(m_n^{(l)})^{1/2}}{\widehat{\sigma}_n^{(l)}}\Big(\frac{1}{m_n^{(l)}}\sum_{j=1}^{m_n^{(l)}}\big(\Delta_{n,j}^{(l)} - \mathbb{E}(\Delta_{n,j}^{(l)}|\mathcal{E}_n)\big)\Big),$$

$$T_{n,2}^{(l)} = \frac{(m_n^{(l)})^{1/2}}{\widehat{\sigma}_n^{(l)}}\Big(R(\widehat{f}_n) - R(f^*) - \big(R_{\mathcal{S}}(\widehat{g}_n) - R_{\mathcal{S}}(g^*)\big)\Big),$$

$$T_{n,3}^{(l)} = \frac{(m_n^{(l)})^{1/2}}{\widehat{\sigma}_n^{(l)}}\big(R(f^*) - R_{\mathcal{S}}(g^*)\big),$$

where $m_n^{(l)} = m_n$ if $l=1$ and $m_n^{(l)} = m_n/2$ if $l=2$, and $T_{n,3}^{(1)} = T_{n,3}^{(2)} = 0$ under H_0.

Now consider $T_{n,1}^{(l)}$ and $T_{n,2}^{(l)}$ separately. To proceed, we first show that $\widehat{\sigma}_n^{(1)} \xrightarrow{p} \sigma^{(1)}$ and $\widehat{\sigma}_n^{(2)} \xrightarrow{p} \sigma^{(2)}$. Specifically, for $l=1,2$,

$$\begin{aligned}
(\widehat{\sigma}_n^{(l)})^2 &= \frac{m_n^{(l)}}{m_n^{(l)}-1}\Big(\frac{1}{m_n^{(l)}}\sum_{j=1}^{m_n^{(l)}}(\Delta_{n,j}^{(l)})^2 - \big(\frac{1}{m_n^{(l)}}\sum_{j=1}^{m_n^{(l)}}\Delta_{n,j}^{(l)}\big)^2\Big)\\
&= \frac{m_n^{(l)}}{m_n^{(l)}-1}\Big(\frac{1}{m_n^{(l)}}\sum_{j=1}^{m_n^{(l)}}\big((\Delta_{n,j}^{(l)})^2 - \mathbb{E}\big((\Delta_{n,j}^{(l)})^2|\mathcal{E}_n\big)\big)\Big)\\
&+ \frac{m_n^{(l)}}{m_n^{(l)}-1}\Big(\big(\frac{1}{m_n^{(l)}}\sum_{j=1}^{m_n^{(l)}}(\mathbb{E}(\Delta_{n,j}^{(l)}|\mathcal{E}_n) - \Delta_{n,j}^{(l)})\big)\\
&\qquad\big(\frac{1}{m_n^{(l)}}\sum_{j=1}^{m_n^{(l)}}(\mathbb{E}(\Delta_{n,j}^{(l)}|\mathcal{E}_n) + \Delta_{n,j}^{(l)})\big)\Big)\\
&+ \frac{m_n^{(l)}}{m_n^{(l)}-1}\Big(\mathrm{Var}(\Delta_n^{(l)}|\mathcal{E}_n)\Big) \xrightarrow{p} (\sigma^{(l)})^2,
\end{aligned}$$

which follows from the continuous mapping theorem, Assumption C for the one-split test or Assumption C′ for the two-split test, and the fact that

$$\frac{1}{m_n^{(l)}}\sum_{j=1}^{m_n^{(l)}}\big(\Delta_{n,j}^{(l)} - \mathbb{E}(\Delta_{n,j}^{(l)}|\mathcal{E}_n)\big) \xrightarrow{p} 0,$$

$$\frac{1}{m_n^{(l)}}\sum_{j=1}^{m_n^{(l)}}\big((\Delta_{n,j}^{(l)})^2 - \mathbb{E}((\Delta_{n,j}^{(l)})^2|\mathcal{E}_n)\big) \xrightarrow{p} 0,$$

which are obtained from the law of large number of the triangular array $\{\Delta_{n,j}^{(l)}\}_{1\le j\le m_n}$ and Assumptions B and B′, c.f., Corollary 9.5.6 of [8].

Consequently, when $m = o(n^{2\gamma})$, it follows from Assumption A and $\widehat{\sigma}_n^{(l)} \xrightarrow{p} \sigma^{(l)} > 0$ that

$$T_{n,2}^{(l)} = \frac{\sqrt{m_n}}{\widehat{\sigma}_n^{(l)}}\Big(R(\widehat{f}_n) - R(f^*) - \big(R_{\mathcal{S}}(\widehat{g}_n) - R_{\mathcal{S}}(g^*)\big)\Big) \xrightarrow{p} 0.$$

Moreover,

$$T_{n,1}^{(l)} = \frac{\sigma^{(l)}}{\widehat{\sigma}_n^{(l)}}\frac{1}{(m_n^{(l)})^{1/2}\sigma^{(l)}}\Big(\sum_{j=1}^{m_n^{(l)}}\big(\Delta_{n,j}^{(l)} - \mathbb{E}(\Delta_{n,j}^{(l)}|\mathcal{E}_n)\big)\Big) \xrightarrow{d} N(0,1), \tag{D.1}$$

which follows from the continuous mapping theorem, Slutsky's Lemma, and the central limit Theorem of the triangular array $\{\Delta_{n,j}^{(l)}\}_{1\le j\le m_n}$, and Assumptions B or B′, c.f., Corollary 9.5.11 of [8]. The desired result then follows. This completes the proof. □

Proof of Theorems 2 and A.2. For q-order combined tests, let $A = \big\{\bar{P}^{(l)} \le \alpha\big\}$ and $B = \big\{\sum_{u=1}^U \mathbb{I}(P_u^{(l)} \le \frac{q\alpha}{U}) \ge q\big\}$. Since $A = \big\{P_{(q)}^{(l)} \le \frac{q\alpha}{U}\big\} = B$, by Markov's inequality, it follows from the assumptions that either Theorem 1 or Theorem A.1 holds. Hence,

$$\begin{aligned}
\mathbb{P}(A|H_0) = \mathbb{P}(B|H_0) &\le \frac{\sum_{u=1}^U \mathbb{P}\big(P_u^{(l)} \le \frac{q\alpha}{U}|H_0\big)}{q}\\
&\to \frac{U\mathbb{P}\big(\Phi(Z) \le \frac{q\alpha}{U}\big)}{q} = \alpha, \ \text{as } n\to\infty,
\end{aligned}$$

where Z follows $N(0,1)$, $\Phi(Z)$ follows the uniform distribution on $[0,1]$, and the last equality follows from continuous mapping theorem.

For Hommel combined test, according to the proof of 3.3 in [9], when $n\to\infty$, we have

$$\begin{aligned}
\mathbb{P}\big(\bar{P}^{(l)} \le \alpha|H_0\big) &\le \sum_{i=1}^{U-1}\frac{1}{i(i+1)}\sum_{u=1}^U \mathbb{P}\big(P_u^{(l)} \le \frac{\alpha i}{C_U U}|H_0\big)\\
&+ \frac{1}{U}\sum_{u=1}^U\big(P_u^{(l)} \le \frac{\alpha}{C_U}|H_0\big) \to \alpha.
\end{aligned}$$

This completes the proof. □

Proof of Theorems 3.1 and A.3. Let $\delta^{(1)} = \delta$ and $\delta^{(2)} = \delta/\sqrt{2}$, using the same argument in the proof of Theorems 1 and A.1, we have that $T_{n,2}^{(l)} \xrightarrow{p} 0$ when $m = o(n^{2\gamma})$, and $T_{n,1}^{(l)} \xrightarrow{d} N(0,1)$. Note that $T_{n,3}^{(l)} = \frac{\sqrt{m_n^{(l)}}}{\widehat{\sigma}_n^{(l)}}\big(R(f^*) - R_{\mathcal{S}}(g^*)\big) = -\delta^{(l)}/\widehat{\sigma}_n^{(l)}$. By Slutsky's theorem, we have

$$\Lambda_n^{(1)} \xrightarrow{d} N(-\delta^{(1)}/\sigma^{(1)}, 1), \quad \Lambda_n^{(2)} \xrightarrow{d} N(-\delta^{(2)}/\sigma^{(2)}, 1). \tag{D.2}$$

Consequently, denote $\beta_n^{(1)}(\delta) = \beta_n(\delta)$, we have

$$\lim_{n\to\infty}\inf \beta_n^{(l)}(\delta) = \Phi\big(z_\alpha - \frac{\delta^{(l)}}{\sigma^{(l)}}\big); \quad l=1,2.$$

The desired result then follows. This completes the proof. □

Proof of Theorems 3.2 and A.4. Denote $\delta^{(1)} = \delta$ and $\delta^{(2)} = \delta/\sqrt{2}$. We first prove for q-order combined tests. Note that $\bar{\pi}_n^{(l)}(\delta) = 1 - \bar{\beta}_n^{(l)}(\delta)$. By Markov's inequality, Type II error is upper bounded by $\mathbb{P}\big(\bar{P}^{(l)} \ge \alpha|H_a\big) \le \min\Big(\frac{U}{\alpha q}\mathbb{E}\big(P_{(q)}^{(l)}|H_a\big), 1\Big)$. To bound the expectation of an order

statistic based on dependent samples, we apply (1) of [10], which is a version of [11]:

$$\mathbb{E}\big(P_{(q)}^{(l)}|H_a\big) \le \bar{\mu} + \Big(\frac{q-1}{U-q+1}\sum_{u=1}^{U}\big(\sigma_u^2 + (\mu_u - \bar{\mu})^2\big)\Big)^{1/2}, \tag{D.3}$$

where $\bar{\mu} = U^{-1}\sum_{u=1}^{U}\mu_u$, $\mu_u = \mathbb{E}(P_u^{(l)}|H_a)$ and $\sigma_u^2 = \mathrm{Var}(P_u^{(l)}|H_a)$.

For $u = 1, \cdots, U$, by (D.2) and Portmanteau's theorem, $\mathbb{E}\big(P_u^{(l)}|H_a\big) \to \mathbb{E}\big(\Phi\big(Z - \frac{\delta^{(l)}}{\sigma^{(l)}}\big)\big)$ as $n \to \infty$. By Corollary 1 of [12], $\mathbb{E}\big(\Phi\big(Z - \frac{\delta^{(l)}}{\sigma^{(l)}}\big)\big) = \Phi\big(-\frac{\delta^{(l)}}{\sqrt{2}\sigma^{(l)}}\big)$. Similarly, $\mathrm{Var}\,\big(P_u^{(l)}|H_a\big) \to \mathrm{Var}\,\big(\Phi\big(Z - \frac{\delta^{(l)}}{\sigma^{(l)}}\big)\big)$; $u = 1, \cdots, U$; $\mathrm{Var}\,\big(\Phi\big(Z - \frac{\delta^{(l)}}{\sigma^{(l)}}\big)\big) = \Phi\big(-\frac{\delta^{(l)}}{\sqrt{2}\sigma^{(l)}}\big) - \Phi^2\big(-\frac{\delta^{(l)}}{\sqrt{2}\sigma^{(l)}}\big) - 2T(-\frac{\delta^{(l)}}{\sqrt{2}\sigma^{(l)}}, \frac{\sqrt{3}}{3})$, where $T(\cdot,\cdot)$ is Owen's T function [1].

Therefore, by (D.3), as $n \to \infty$,

$$\begin{aligned}\mathbb{E}\big(P_u^{(l)}|H_a\big) &\to \mathbb{E}\big(\Phi\big(Z - \frac{\delta^{(l)}}{\sigma^{(l)}}\big)\big) \\ &\quad + \Big(\frac{q-1}{U-q+1}\,\mathrm{Var}\,\big(\Phi\big(Z - \frac{\delta^{(l)}}{\sigma^{(l)}}\big)\big)\Big)^{1/2} \\ &= \Phi\big(-\frac{\delta^{(l)}}{\sqrt{2}\sigma^{(l)}}\big) \\ &\quad + \sqrt{\frac{q-1}{U-q+1}}\Big(\Phi\big(\frac{\delta^{(l)}}{\sqrt{2}\sigma^{(l)}}\big) - \Phi^2\big(\frac{\delta^{(l)}}{\sqrt{2}\sigma^{(l)}}\big) \\ &\quad - 2T(-\frac{\delta^{(l)}}{\sqrt{2}\sigma^{(l)}}, \frac{\sqrt{3}}{3})\Big)^{1/2}.\end{aligned}$$

The desired result then follows. Therefore, for Hommel combined test,

$$\mathbb{P}\big(C_U \min_{1\le q\le U}\frac{U}{q}P_{(q)}^{(l)} \le \alpha|H_a\big) \ge \max_{1\le q\le U}\mathbb{P}\big(\frac{U}{q}P_{(q)}^{(l)} \le \frac{\alpha}{C_U}|H_a\big).$$

The desired result then follows by taking limits for both sides. This completes the proof. □

Proof of Lemma C.1. To proceed, let $f_0 \in \mathcal{H}$ be a neural network, and its weight matrix is exactly same with that of g_0 defined in (C.1), expect that j-th ($j \in \mathcal{S}_0$) column in the first layer is set as zero, which implies that $f^*(\boldsymbol{X}) = f_0(\boldsymbol{X}) = g_0(\boldsymbol{Z}(\boldsymbol{X}))$.

To verify Assumption A, it suffices to verify the entropy condition of $\mathcal{H}$. By Theorem I of [13], for any $\omega > 0$, we have

$$\log\mathcal{N}\big(u, \mathcal{H}, \|\cdot\|_2\big) \le c(p, \varpi, \tau, L)\log(u^{-1}) = O(u^{-\omega}),$$

where $\mathcal{N}\big(u, \mathcal{H}, \|\cdot\|_2\big)$ is the covering number based on L_2-norm, $c(p, \varpi, \tau, L)$ is a constant depends on p, ϖ, τ, and L. By [14],

$$\begin{aligned} R(\widehat{f}_n) - R(f^*) &= \mathbb{E}\big(\widehat{f}_n(\boldsymbol{X}) - f^*(\boldsymbol{X})\big)^2 = O_p(n^{-1+\omega}), \\ R_{\mathcal{S}}(\widehat{g}_n) - R_{\mathcal{S}}(g^*) &= \mathbb{E}\big(\widehat{g}_n(\boldsymbol{X}) - g^*(\boldsymbol{X})\big)^2 = O_p(n^{-1+\omega}). \end{aligned} \tag{D.4}$$

Therefore, $\gamma = 1 - \omega$ for Assumption A with any $\omega > 0$. Then Assumptions B and B$'$ follow from the fact that $f \in \mathcal{H}$ is upper bounded by a constant, that is

$$\begin{aligned}\sup_{\boldsymbol{x}\in[-1,1]^d}|f(\boldsymbol{x})| &= \sup_{\boldsymbol{x}\in[-1,1]^d}\big|A\big(\boldsymbol{W}^L\cdots A(\boldsymbol{W}^1\boldsymbol{x})\big)\big| \\ &\le \Big(\prod_{l=1}^{L}\|\boldsymbol{W}^l\|_2\Big)\sup_{\boldsymbol{x}\in[-1,1]^d}\|\boldsymbol{x}\|_2 \le \sqrt{p}\tau^L,\end{aligned} \tag{D.5}$$

where the second last inequality follows from the definition of the matrix norm. Next, we verify Assumptions C and C$'$. Let $\mathbb{E}_n(\cdot) = \mathbb{E}(\cdot|\mathcal{E}_n)$, $\mathrm{Var}_n(\cdot) = \mathrm{Var}(\cdot|\mathcal{E}_n)$, $\Psi(f, g, \boldsymbol{U}) = l(f(\boldsymbol{X}), Y) - l(g(\boldsymbol{Z}(\boldsymbol{X})), Y)$, and $\boldsymbol{U} = (\boldsymbol{X}, Y)$. Then,

$$\begin{aligned}\mathrm{Var}_n(\Delta_{n,1}^{(1)}) &= \mathrm{Var}_n\big(\Psi(\widehat{f}_n, \widehat{g}_n, \boldsymbol{U})\big) + \rho_n^2 \\ &= \mathrm{Var}_n\big(\Psi(\widehat{f}_n, f^*, \boldsymbol{U}) + \Psi(f^*, g^*, \boldsymbol{U}) \\ &\quad + \Psi(g^*, \widehat{g}_n, \boldsymbol{U})\big) + \rho_n^2 \\ &= \mathrm{Var}_n\big(\Psi(\widehat{f}_n, \widehat{g}_n, \boldsymbol{U})\big) + \mathrm{Var}_n\big(\Psi(f^*, g^*, \boldsymbol{U})\big) \\ &\quad + \mathrm{Var}_n\big(\Psi(g^*, \widehat{g}_n, \boldsymbol{U})\big) \\ &\quad + 2\,\mathrm{Cov}\,\big(\Psi(\widehat{f}_n, \widehat{g}_n, \boldsymbol{U}), \Psi(f^*, g^*, \boldsymbol{U})\big) \\ &\quad + 2\,\mathrm{Cov}\,\big(\Psi(\widehat{f}_n, \widehat{g}_n, \boldsymbol{U}), \Psi(g^*, \widehat{g}_n, \boldsymbol{U})\big) \\ &\quad + 2\,\mathrm{Cov}\,\big(\Psi(f^*, g^*, \boldsymbol{U}), \Psi(g^*, \widehat{g}_n, \boldsymbol{U})\big) + \rho_n^2 \\ &\xrightarrow{p} \mathrm{Var}\big(\Psi(f^*, g^*, \boldsymbol{U})\big) + \rho_n^2 = (\sigma^{(1)})^2,\end{aligned}$$

where the last equality follows from the uniform boundedness of $\mathcal{H}$ in (D.5) and the fact that $\mathrm{Var}_n\big(\Psi(g^*, \widehat{g}_n, \boldsymbol{U})\big), \mathrm{Var}_n\big(\Psi(\widehat{f}_n, f^*, \boldsymbol{U})\big) \xrightarrow{p} 0$. Specifically,

$$\begin{aligned}\mathrm{Var}_n\big(\Psi(\widehat{f}_n, f^*, \boldsymbol{U})\big) &\le \mathbb{E}_n\big(\Psi^2(\widehat{f}_n, f^*, \boldsymbol{U})\big) \\ &= \mathbb{E}_n\Big(\big(\widehat{f}_n(\boldsymbol{X}) - f^*(\boldsymbol{X})\big)^2\big(\widehat{f}_n(\boldsymbol{X}) + f^*(\boldsymbol{X}) - 2Y\big)^2\Big) \\ &\le (2\sqrt{p}\tau + 4\varsigma^2)\mathbb{E}_n\Big(\big(\widehat{f}_n(\boldsymbol{X}) - f^*(\boldsymbol{X})\big)^2\Big) \\ &= (2\sqrt{p}\tau + 4\varsigma^2)\big(R(\widehat{f}_n) - R(f^*)\big) \xrightarrow{p} 0.\end{aligned}$$

Similarly, we can show that $\mathrm{Var}_n\big(\Psi(g^*, \widehat{g}_n, \boldsymbol{U})\big) \xrightarrow{p} 0$.

Moreover, for $\Delta_n^{(2)}$, using the same argument, we have

$$\begin{aligned}\mathrm{Var}_n\big(\Delta_n^{(2)}\big) &= \mathrm{Var}_n\big(l(\widehat{f}_n(\boldsymbol{X}), Y) - l(\widehat{g}_n(\boldsymbol{Z}'), Y')\big) \\ &= \mathrm{Var}_n\big(l(\widehat{f}_n(\boldsymbol{X}), Y)\big) + \mathrm{Var}_n\big(l(\widehat{g}_n(\boldsymbol{Z}), Y)\big) \\ &\xrightarrow{p} \mathrm{Var}(l(f^*(\boldsymbol{X}), Y)) + \mathrm{Var}(l(g^*(\boldsymbol{Z}), Y)) = (\sigma^{(2)})^2.\end{aligned}$$

The desired result then follows. This completes the proof. □

Proof of Lemma 2. By the definitions of n and m in Section 3.1, we have

$$m = N - n \le N - x_0 = N_0\log(x_0)/2/\log(N_0/2) = o(n^{2\gamma}),$$

where $\gamma > 0$ is any fixed constant in Assumption A, and the last equality follows from $n \ge x_0$. This completes the proof. □

REFERENCES

[1] D. B. Owen, "Tables for computing bivariate normal probabilities," *Annals of Mathematical Statistics*, vol. 27, no. 4, pp. 1075–1090, 1956.

[2] R. Ge, F. Huang, C. Jin, and Y. Yuan, "Escaping from saddle points—online stochastic gradient for tensor decomposition," in *Conference on Learning Theory*, 2015, pp. 797–842.

[3] M. Raginsky, A. Rakhlin, and M. Telgarsky, "Non-convex learning via stochastic gradient langevin dynamics: a nonasymptotic analysis," in *Conference on Learning Theory*. PMLR, 2017, pp. 1674–1703.

[4] L. Wu, C. Ma, and E. Weinan, "How sgd selects the global minima in over-parameterized learning: A dynamical stability perspective," in *Advances in Neural Information Processing Systems*, 2018, pp. 8279–8288.

[5] D. F. McCaffrey and A. R. Gallant, "Convergence rates for single hidden layer feedforward networks," *Neural Networks*, vol. 7, no. 1, pp. 147–158, 1994.

[6] M. Kohler and A. Krzyżak, "Adaptive regression estimation with multilayer feedforward neural networks," *Nonparametric Statistics*, vol. 17, no. 8, pp. 891–913, 2005.

[7] M. Kohler and A. Krzyzak, "Nonparametric regression based on hierarchical interaction models," *IEEE Transactions on Information Theory*, vol. 63, no. 3, pp. 1620–1630, 2016.

[8] O. Cappé, E. Moulines, and T. Rydén, *Inference in Hidden Markov Models*. Springer Science & Business Media, 2006.

[9] G. Hommel, "Tests of the overall hypothesis for arbitrary dependence structures," *Biometrical Journal*, vol. 25, no. 5, pp. 423–430, 1983.

[10] D. Bertsimas, K. Natarajan, and C.-P. Teo, "Tight bounds on expected order statistics," *Probability in the Engineering and Informational Sciences*, vol. 20, no. 4, p. 667, 2006.

[11] B. C. Arnold, R. A. Groeneveld *et al.*, "Bounds on expectations of linear systematic statistics based on dependent samples," *Annals of Statistics*, vol. 7, no. 1, pp. 220–223, 1979.

[12] B. E. Ellison, "Two theorems for inferences about the normal distribution with applications in acceptance sampling," *Journal of the American Statistical Association*, vol. 59, no. 305, pp. 89–95, 1964.

[13] Z.-C. Guo, L. Shi, and S.-B. Lin, "Realizing data features by deep nets," *IEEE Transactions on Neural Networks and Learning Systems*, vol. 31, no. 10, pp. 4036–4048, 2019.

[14] Q. Han, J. A. Wellner *et al.*, "Convergence rates of least squares regression estimators with heavy-tailed errors," *Annals of Statistics*, vol. 47, no. 4, pp. 2286–2319, 2019.
